# EEG hybrid brain-computer interfaces: A scoping review applying an existing hybrid-BCI taxonomy and considerations for pediatric applications

**DOI:** 10.3389/fnhum.2022.1007136

**Published:** 2022-11-17

**Authors:** Matheus G. Mussi, Kim D. Adams

**Affiliations:** Assistive Technology Laboratory, Faculty of Rehabilitation Medicine, University of Alberta, Edmonton, AB, Canada

**Keywords:** hybrid, BCI, scoping review, EEG, children, clinical

## Abstract

Most hybrid brain-computer interfaces (hBCI) aim at improving the performance of single-input BCI. Many combinations are possible to configure an hBCI, such as using multiple brain input signals, different stimuli or more than one input system. Multiple studies have been done since 2010 where such interfaces have been tested and analyzed. Results and conclusions are promising but little has been discussed as to what is the best approach for the pediatric population, should they use hBCI as an assistive technology. Children might face greater challenges when using BCI and might benefit from less complex interfaces. Hence, in this scoping review we included 42 papers that developed hBCI systems for the purpose of control of assistive devices or communication software, and we analyzed them through the lenses of potential use in clinical settings and for children. We extracted taxonomic categories proposed in previous studies to describe the types of interfaces that have been developed. We also proposed interface characteristics that could be observed in different hBCI, such as type of target, number of targets and number of steps before selection. Then, we discussed how each of the extracted characteristics could influence the overall complexity of the system and what might be the best options for applications for children. Effectiveness and efficiency were also collected and included in the analysis. We concluded that the least complex hBCI interfaces might involve having a brain inputs and an external input, with a sequential role of operation, and visual stimuli. Those interfaces might also use a minimal number of targets of the strobic type, with one or two steps before the final selection. We hope this review can be used as a guideline for future hBCI developments and as an incentive to the design of interfaces that can also serve children who have motor impairments.

## 1. Introduction

Children with very limited motor abilities may benefit from the use of brain-computer interfaces (BCI) to access play and learning activities, but there is very little research in the area. BCI are devices that use brain signals processed *via* computational operations to control machines for various purposes, from rehabilitation to gaming. Despite the long list of interfaces created to this day, most traditional BCI still face challenges in achieving the desired performance needed for reliably controlling assistive devices. Hybrid brain-computer interfaces (hBCI) may be able to address the limitations of traditional single-input BCI (Kinney-Lang et al., 2020; Orlandi et al., 2021). The main goal of hBCI is to improve BCI performance through multi-modal signal inputs, e.g., combinations of different brain signals, BCI paradigms, and/or other external devices (Wolpaw and Wolpaw, 2012).

There have been a few reviews of traditional single-input BCI use with children (Mikołajewska and Mikołajewski, 2014; Beraldo et al., 2020; Orlandi et al., 2021). However, the reviews only revealed 8 unique studies for BCI control of devices, and no reviewed studies used hBCI. In a review by Karlsson et al. (2022), hybrid BCIs are mentioned as a potential technology for children with disabilities to attain better accuracies and reduce errors, however, only studies with adults were cited.

There have been several reviews on hBCI but they primarily included studies that tested systems with adults who do not have disabilities: Sharmila (2020) provided an overview on the types of hBCI for wheelchair-based systems; Neeling and Hulle (2019) focused on multi-input hybrids and their applications; Sadeghi and Maleki (2018) compared accuracy and information transfer rate (ITR) across systems; Hong and Khan (2017) discussed the combination of brain signals and their application for both clinical and non-clinical scenarios; Choi et al. (2017) did a systematic review and proposed a taxonomy classification system for hBCI systems; Banville and Falk (2016) did a systematic review and discussed experimental protocols, signal processing, and study rational; and Amiri et al. (2013) reviewed multi-brain signal hBCIs. Muller-Putz et al. (2015) compared hBCI applications that had participants with and without disabilities. There have also been studies using hBCI that proposed taxonomies or summarized it, such as Li et al. (2019) who categorized hBCI according to Multiple Brain Patterns, Multi-sensory and Multiple Signals, and Allison et al. (2011) who summarized the initial efforts in hybridization and the perspectives of hBCI.

BCI for children differs from implementation for adults for several reasons. There might be difficulties in identifying signal features (Mikołajewska and Mikołajewski, 2014), recognizing oscillatory brain signals (Ehlers et al., 2012), and instructing young participants to perform the desired self-regulating mental task (Zhang et al., 2019b). During experiments, external factors such as lab environment or the presence of the caregiver can distract children and negatively influence the recorded signal (Richards, 2003; de Haan, 2007; Gavin and Davies, 2007). In addition, BCI system requirements may be difficult, overwhelming or unpleasant to the children, causing them to lose interest or be unable to continue (Gavin and Davies, 2007). Cognitive skills that affect BCI performance are beginning to be explored with adults, such as working memory, general intelligence, executive function (Sprague et al., 2016), maintaining attention (Riccio et al., 2013), and task switching (Pitt and Brumberg, 2018). The Beraldo review summarized that children 11 years and older achieved performance comparable to adults (Beraldo et al., 2020). However, some of the needed cognitive skills are only apparent after a certain level of brain development (Menary et al., 2013), for example, Cowan et al. (2006) reported that children have poorer attention capabilities than adults.

The reviews on hBCI covered a wide variety of applications and analyses, but it is difficult to determine what might be appropriate to develop for use with children. Therefore, the following is a review of the state of the art in hBCI, including factors that may affect ease of use.

## 2. Review objectives

The objective of this review was to examine the literature around hBCI with regards to clinical applications, especially applied to control of devices and communication, with a lens for potential use in the pediatric population (less than 18 years old). The guiding research questions were: (1) What are the existing approaches for hBCI systems that are focused on control of devices, that could be used clinically?; (2) What are the factors of the hBCI that may influence use by children?

## 3. Search methodology

The keyword search consisted of four parts to specify the hybrid modality, the BCI system, the application type and the acquisition source: (Hybrid* OR Multi-input* OR Multi-Sensor* OR Multi-Device* OR Multimodal) ***AND*** (“brain computer interface” OR BCI* OR hBCI OR “human machine interface” OR HMI) ***AND*** (Activit* OR Task* OR Step* OR Assignment* OR Exercise OR Test* OR Execut*) ***AND*** (((EEG) OR (electroencephalogra*)) OR (non-invasive)).

As the definition of a hybrid BCI (hBCI) can be broad, to narrow down our scope, we defined some minimal requirements for a hBCI system to be considered valid for our analysis. **Firstly**, we only considered systems that included signal acquisition *via* electroecephalography (EEG). EEG-based BCI are the most popular system compared to other non-invasive methods [such as near infra-red (NIRS), functional NIRS (fNIRS), or magnetoencephalography] and they have the highest information transfer rates (ITR), which puts them in an advantageous position in terms of performance compared to other methods. **Secondly**, the BCI component must have had a primary role in the overall system. Counterexamples of this requirement would be hBCI that used brain signals only for target selection confirmation or hBCI that used brain signals only as a mechanism to switch between non-BCI input modes. **Thirdly**, the multiplicity of inputs or paradigms had to work synergistically to achieve improved results. The main interest in this review was in systems that combine different paradigms, inputs or sensory pathways attempting to improve traditional BCI. Systems that implemented two paradigms to execute completely unrelated tasks, although they happen to be accessed through the same interface, were not considered for this review. Systems that combined BCI paradigms and a switch mechanism to perform separate tasks were not considered. For example, a system that used one BCI paradigm to move a wheelchair and another to select items on a shelf were rejected. Likewise, systems that integrated an on/off switch mechanism to a previously standalone BCI were also rejected. We understood that such systems did not significantly contribute to the improvement of the system's performance but rather with its controllability and asynchronous capabilities.

The scoping review methodology proposed by Arksey and O'Malley (2005) was implemented. Articles from Web of Science, PubMed, Scopus, and IEEE Xplore databases were extracted as they focus on medical and engineering topics. The criteria for inclusion and exclusion were delimited per filtering phase, following the scoping review methodology. The exclusion criteria of the previous phases were kept for the next phases in case the article did not explicitly mention an exclusive term in the previous phase. All databases were searched on February 23^rd^ of 2021, and articles published before that date were included without specific cutoff criteria. Patents, reviews, and other formats of publication that were not articles or conference papers were not included.

### 3.1. Title inclusion/exclusion criteria

Article titles to be *included* had to: (1) contain “hybrid BCI” or other terms that indicated hybridization such as multi-input, multi-modality or multiple paradigms, signal acquisition methods or devices; and (2) make reference to control terms (selection, interaction, classification, etc.) or devices (speller, robotic arm, wheelchair, etc.). The titles that were *excluded* were the ones indicating that the paper focused on: (1) non-hBCI systems (e.g., single-input BCI, multi-input devices), (2) estimation applications (e.g., motion trajectory prediction, group decision making), (3) assessment applications (learning performance, affective/emotion state, mental/psychological state, facial expressions or fatigue), (4) imaging and detection applications (e.g., neuroimaging, algorithms to localize best EEG sources, studies on brain signal detection, cortical reorganization, epilepsy detection), (5) other EEG-related algorithms, (e.g., artifact removal algorithms, algorithm for EEG signal simulation), (6) rehabilitation or therapeutic applications, (7) systems including functional electrical stimulation, (8) invasive technologies, and (9) pure headset development.

### 3.2. Abstract exclusion criteria

At this stage, the title-included articles were filtered based on their abstracts. Articles were excluded if they were oriented toward: (1) BCI as a complementary input in a multi-modal system, (2) the study of hybrid classifiers for a single BCI input, (3) optimal channel selection algorithms, (4) development of a method or framework for experimentation, (5) signal identification during other activities or (6) if they had no participants (i.e., only used datasets for validation).

### 3.3. Full article exclusion criteria

The final filter allowed a more in-depth analysis of each article. At this phase, articles were excluded if they: (1) proposed an invalid hBCI (i.e., brain signal was a secondary function, any of the inputs in a two-system input was only used to keep/turn the system on/off, paradigms or inputs did not work in synergy), (2) did not have a valid performance measurement (accuracy or true positives, true negatives, false positives and false negatives, or any indication of the number of correct trials vs. the total) of the paradigms' efficiency (as opposed to the task accomplishment, which was not the main interest), (3) had online trials but only provided offline performance measurements, (4) did not include performance measurements for the relevant system role of operation, either for each of the inputs or their combination, (5) proposed a system that was not suitable for control applications, (6) had insufficient information for experiment replicability (i.e., lacked information such as, but not limited to, number of trials, number of participants, number of sessions, number of analyzed samples, number of training/validation datasets, or clarity about experiment protocol).

## 4. Data extraction

Descriptive information was extracted from the articles such as study population, size, age, control task, and the tools that were used for the development of the hBCI (model, programming languages, toolboxes). System data was extracted and labeled according to the hBCI taxonomy proposed by Choi et al. (2017) (see Table 1). The categories used were: (1) diversity of input signal, (2) role of operation, (3) mode of operation, (4) mental strategy, (5) brain signal signature, and (6) stimulus modality. A brief description of the categories is provided here:

**Diversity of input** is categorized as homogeneous or heterogeneous, depending on whether the input types are brain signals only, or brain signals combined with other inputs, respectively. Homogeneous hybrid-inputs can have a single-brain signal approach [e.g., both electroencephalography (EEG)] with multiple paradigms or a multi-brain signal approach [e.g., EEG and functional near-infrared spectroscopy (fNIRS)] with single or multiple paradigms. Heterogeneous inputs combine multi-physiological signals [e.g., EEG and electromyography (EMG)] or external inputs (e.g., EEG and Eye Tracker).
**Role of operation** refers to the role of each system and how they are chronologically bound together. The role can be simultaneous, where both systems work concurrently in either the same or in different parts of the task. It can also be a sequential-switch, where one system initiates the other system, or a sequential-selector, where one system partially completes the task and the other system confirms or rejects the selection.
**Mode of operation** is the mode with which the experiment is paced. For synchronous experiments, stimuli are presented within a specific timeframe and cues are used. Asynchronous interfaces are self-paced by the subject, with more flexible timeframes.
**Mental strategy** is categorized as either selective attention or operant conditioning. Selective attention strategies rely on external stimuli to generate expected brain responses, while operant conditioning strategies (also known as slow cortical potentials) rely on the self-regulation of the subject to generate distinguishable brain responses.
**Brain signal signature** are defined by the mental paradigm used for the interface, and is directly associated with the mental strategy. For selective attention, the steady-state evoked potential (SSEP), transient event-related potentials (ERP) and motion-onset evoked potential (mVEP) are possible signatures. For operant conditioning, slow cortical potentials (SCP) can be modulated *via* movement related efforts (sensory-motor rhythms—SMR) or attention levels (μ-rhythm). Other mental tasks involving music and speech imagery were also classified as SCP.
**Stimulus modality** is the pathway through which the user is stimulated so that the brain can elicit predictable signals. The pathway can be sensorial such as visual, tactile or auditory, or self-induced in the case of operant conditioning, defined as the operant pathway. A further classification can be made in terms of diversity of stimulus modalities within the interface. Single modality uses the same sensory pathway for all inputs and paradigms, and multi-modality uses different sensory pathways for the same brain signature [e.g., steady-state visual evoked potential (SSVEP) and steady-state somatosensory evoked potential (SSSEP)].

**Table 1:** Taxonomic features.

**References**	**Diversity of input**	**Role of Op**.	**Mode of Op**.	**Mental strat**.	**Brain sign**.	**Stim. mod**.
Ahn et al. (2014)	SB	Sim	Synch	Op. Cond.	SMR mu	Operant tactile
	SB	Seq	Synch	Op. Cond.	SMR mu	Operant tactile
Allison et al. (2010)	SB	Sim	Synch	Op. cond. Sel. att.	SSEP SMR	Visual
Allison et al. (2012)	SB	Sim	Synch	Op. cond. Sel. att.	SSEP SMR	Visual operant
An et al. (2014)	SB	Seq	Synch	Sel. att.	ERP	Visual auditory
	SB	Sim	Synch	Sel. att.	ERP	Visual auditory
Khalaf et al. (2020)	MB	Sim	Synch	Op. cond.	SMR	Operant
	MB	Sim	Synch	Op. cond. Sel. att.	SSEP SCP	Visual operant
Ko et al. (2020)	SB	Seq	Asynch	Sel. att.	SSEP ERP	Visual
Lee et al. (2018)	MPh	Seq	Synch	Sel. att.	ERP	Visual
	MPh	Seq	Asynch	Sel. att.	ERP	Visual
Li et al. (2018)	SB	Seq	Synch	Op. cond. Sel. att.	SSEP SMR	Visual operant
Lin et al. (2016)	MPh	Sim	Asynch	Sel. att.	SSEP	Visual
	MPh	Sim	Synch	Sel. att.	SSEP	Visual
Long et al. (2012a)	SB	Seq	Asynch	Op. cond. Sel. att.	ERP SMR	Visual operant
Long et al. (2012b)	SB	Sim	Synch	Op. cond. Sel. att.	ERP SMR	Visual operant
Mannan et al. (2020)	EI	Sim	Synch	Sel. att.	SSEP	Visual
Mousavi et al. (2020)	SB	Sim	Synch	Op. cond.	SMR	Operant
Nann et al. (2020)	MPh	Seq	Synch	Op. cond.	SMR	Operant
Saravanakumar and Reddy (2019)	EI	Sim	Synch	Sel. att.	SSEP	Visual
Shi et al. (2019)	MPh	Seq	Asynch	Op. cond.	SMR	Operant
Shin et al. (2018)	MB	Seq	Synch	Op. cond.	SMR SCP	Visual operant
Soekadar et al. (2015)	MPh	Seq	Synch	Op. cond.	SMR	Operant
Wu et al. (2016)	SB	Sim	Synch	Sel. att.	SSEP ERP	Visual
Xu et al. (2014)	SB	Sim	Synch	Sel. att.	SSEP ERP	Visual
Xu et al. (2020)	SB	Sim	Asynch	Sel. att.	SSEP ERP	Visual
	SB	Sim	Synch	Sel. att.	SSEP ERP	Visual
Yang et al. (2020a)	SB	Seq	Synch	Sel. att.	SSEP	Visual
Yang et al. (2020b)	SB	Sim	Synch	Op. cond. Sel. att.	SSEP SMR	Visual Operant
Yao et al. (2014)	SB	Sim	Synch	Op. cond.	SMR	Operant
Yao et al. (2017)	SB	Seq	Synch	Op. cond.	SMR	Operant
Yu et al. (2017)	SB	Seq	Asynch	Op. cond. Sel. att.	ERP SMR	Visual Operant
Yu et al. (2019)	SB	Seq	Synch	Sel. att.	ERP	Visual
Zhang et al. (2019a)	MPh	Seq	Synch, Asynch	Op. cond.	SMR	Operant
Zhou et al. (2020)	MPh	Sim	Asynch	Sel. att.	SSEP	Visual

Underlined elements indicate different experiments within a paper. SB, single-brain signal; MB, multi-brain signal; MPh, multi-physiological; EI, external input; Sim, Simultaneous; Seq, Sequential; Synch, Synchronous; Asynch, Asynchronous; mu, μ-rhythm.

Some interface characteristics that might play an important role in the complexity of a hBCI system were also tracked. These characteristics are important to be considered when designing hBCI as they may directly impact the workload, appeal and the level of engagement of children when using the system.

**Type of target** refers to the kind of stimuli that happens on the screen. Target types can either elicit a certain brain response or indicate to the participant what self-regulating action to take. On-screen targets require visual focus on the stimuli so that the brain can evoke certain signal patterns. The still targets flash periodically (usually with less than 6 Hz) with a certain inter-stimuli interval and are usually associated with P300 paradigms and spellers. Those targets generally require counting and focus on a single desired target. Strobic targets have flashing with higher frequencies (usually above 6 Hz) incorporated into them. They are mostly used in SSEP or rapid serial visual presentation (RSVP) paradigms and can change in intensity, color, shape, visuals or position, and targets usually have different frequencies. Spatial targets stimulate users to displace objects, cursors or other elements over time by triggering a certain threshold of intensity. They are mostly used for slow cortical potentials.
  There are also off-screen targets, which require greater focus and mental training as they do not present stimuli, nor feedback in some cases (Mahmoudi and Erfanian, 2006). These include motor/tactile (MoTa) targets, which require focus on certain motor imageries or tactile stimulations. This approach could be an alternative for people with significant visual impairments. Mental tasks measure the blood flow generated in the brain when arithmetic operations, mental geometric manipulation or word formation are performed by the participant. They are usually associated with NIRS, fNIRS and functional transcranial Doppler ultrasound (fTCD) inputs. Finally, sound cues are targets that rely on sound for selection. These targets can be difficult to distinguish, even when the audio tracks are substantially manipulated as in An et al. (2014) or Glowinsky et al. (2018).
**Number of targets** can vary depending on the application and the tasks to be performed with the interface. Some authors have attempted to increase the number of targets to increase the ITR. Although it might be a good strategy that can give the user more flexibility and a faster system, a greater number of targets could make a user distracted or overwhelmed by many options.
**Number of steps before selection** can also be considered the number of sub-tasks required. Some systems require multiple steps before a final selection is completed (i.e., multiple input commands and classifications needed to make a final selection). Although having multiple steps can add redundancy that can make the final selection more accurate, it could also increase a system's complexity.

Effectiveness and efficiency were the two main performance metrics considered. As most of the BCI community uses accuracy (or other parameters that allow for accuracy assessment) to indicate effectiveness, we used accuracy as our metric for effectiveness. Huggins et al. (2011) reported that accuracies above 90% are expected by potential BCI users. On the other hand, efficiency is measured in a variety of different ways. Most papers that do present efficiency metrics use information transfer rate (ITR), but execution time or commands per minute are also recurrent. It is important to note that our main interest was in the metrics referring to the overall BCI system's classification and not to the task accomplishments.

## 5. Search results

**Figure 1:**
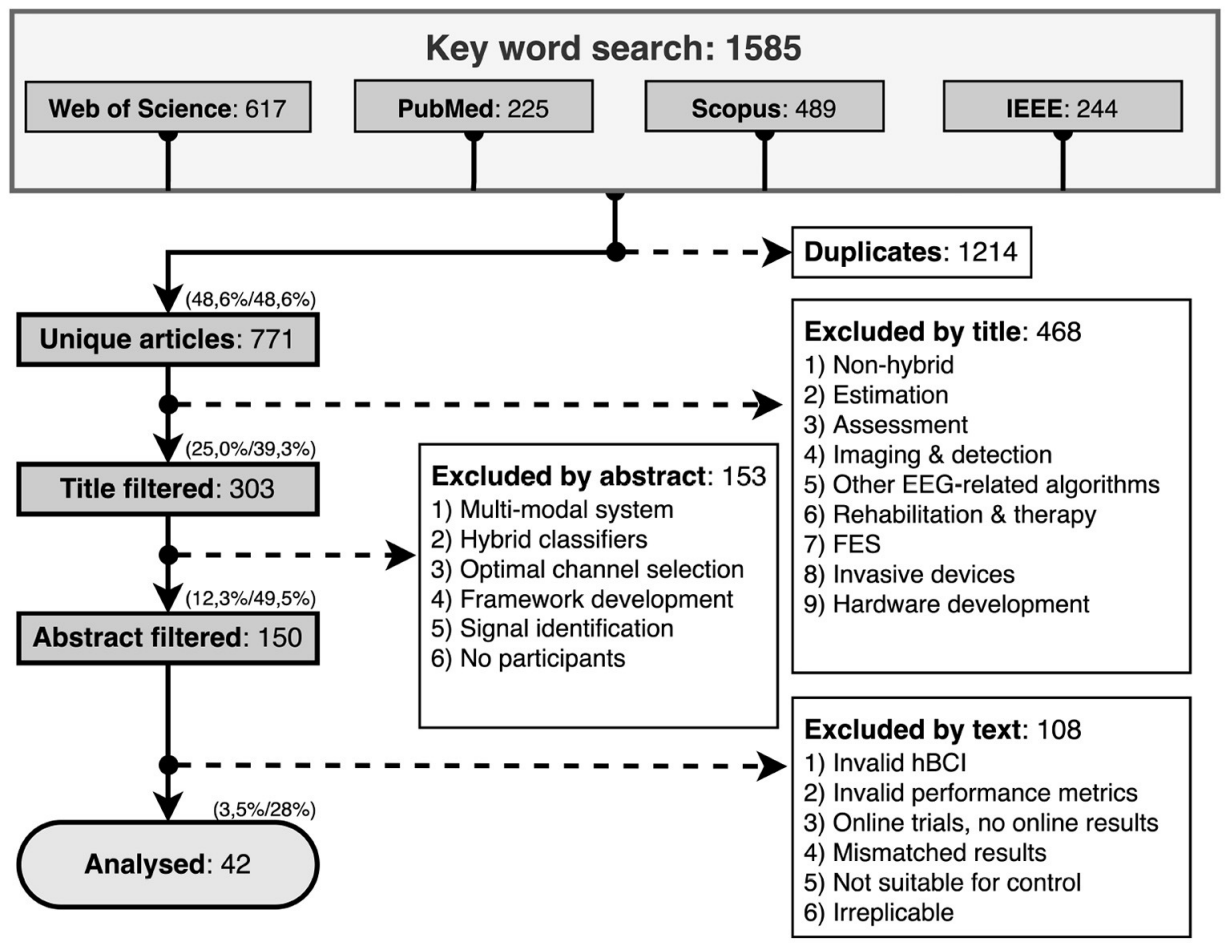
Inclusion/exclusion criteria flowchart.

When all the filters were applied, 42 articles were selected for this scoping review, as shown in Figure 1. Initially, the search on all databases yielded 1,585 publications, 617 from Web of Science, 225 from PubMed, 489 from Scopus, and 244 from IEEE Xplore. The number of duplicates was 1214, which, when removed, resulted in 771 unique articles. From those, 303 were included after title-filtering, 150 after abstract-filtering and 42 after article-filtering. No conference papers remained among the 42 final articles, although we did not set a strict exclusion criteria for conference papers.

**Figure 2:**
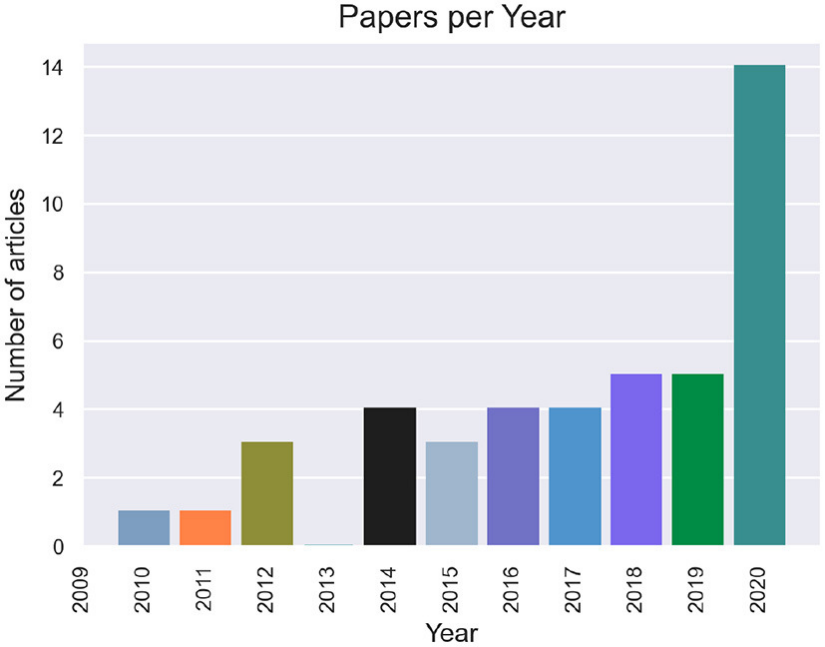
Number of papers included per year.

Figure 2 shows how many articles were published per year. The earliest paper that passed the filtering criteria was from Allison et al. (2010). The overall number of articles per year has been growing. In 2020, 14 articles were written that matched our criteria, over three times more than in the previous six years.

### 5.1. Descriptive data

**Figure 3:**
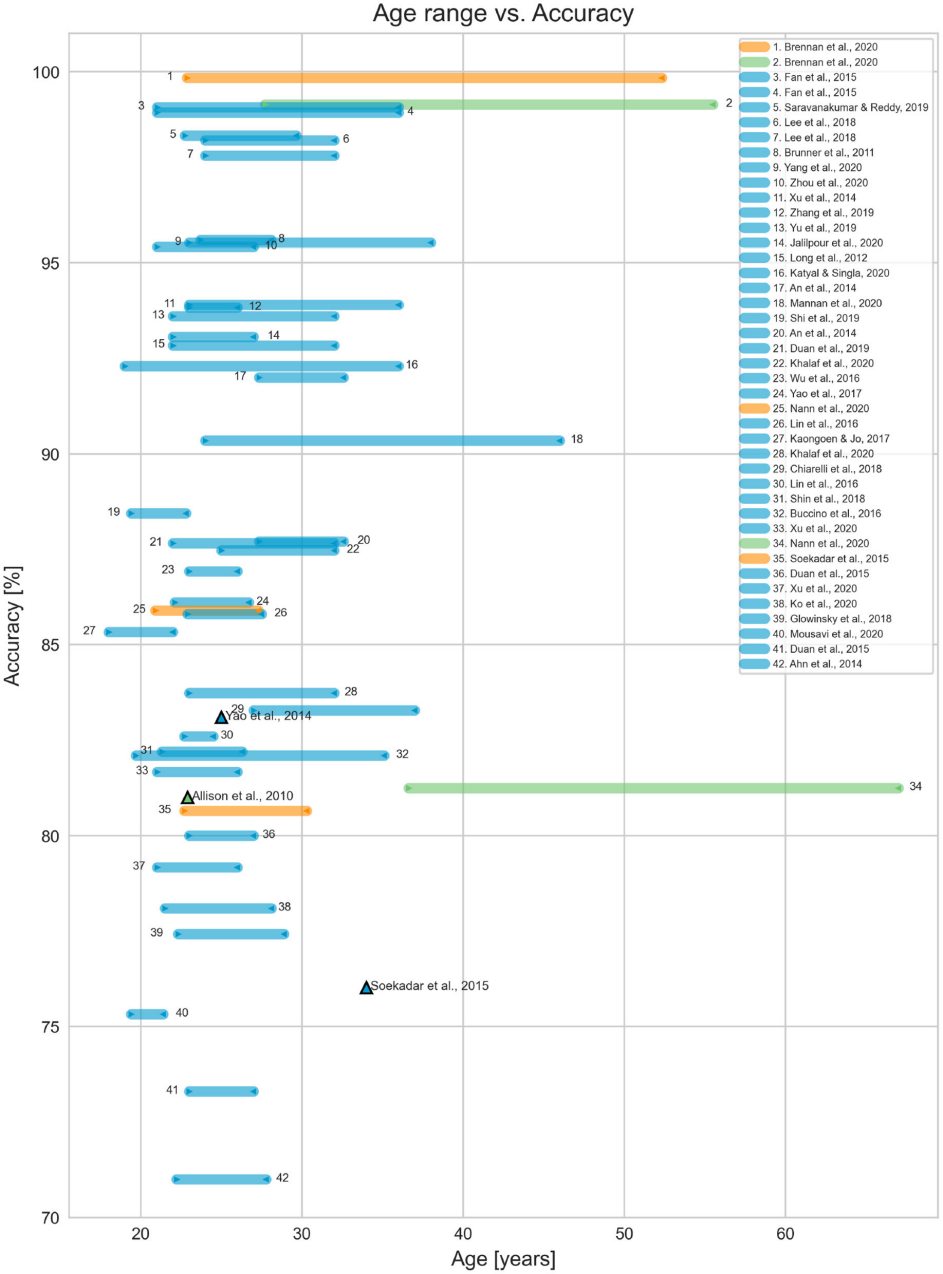
System's accuracy vs. population age. The ranges in blue only included participants without disabilities. The age ranges indicated in green and orange represent the results for participants with and without disabilities within the same paper, respectively. The triangle markers represent system results with a single participant.

Population diagnosis, size and age range are shown in Table 2. No studies included child participants. One study had a participant aged 18 years (Kaongoen and Jo, 2017) but most studies had at least one participant within the range of 20–30 years, except for Nann et al. (2020) who worked with tetraplegic participants aged 51.8 ± 15.2 years. Only two papers included participants above 40 years of age (Brennan et al., 2020; Nann et al., 2020). Figure 3 displays the age range of participants in each study with the achieved average accuracy. Ahn et al. (2014) and Breitwieser et al. (2016) were omitted from the graph because their average accuracies were less than 70% (44.5 and 60%, respectively).

**Table 2:** Performance and interface traits.

**References**	**Pop. Size**	**Pop. age**	**Diagnosis**	**Input 1**	**Input 2**	**Target, types**	**N. targets**	**Steps**	**Control**	**Acc [%]**	**ITR [b/min]**
Ahn et al. (2014)	16	22.22 to 27.78	-	MI	TSA	MoTa	2	1	-	60	-
	16	22.22 to 27.78	-	MI	TSA	MoTA	2	2	-	71	-
Allison et al. (2012)	14	22.9	-	MI	SSVEP	Strobe, MoTA	2	2	-	81	-
Allison et al. (2010)	10	-	-	SSVEP	MI	Strobe, Spatial	8	2	Cursor	60	-
An et al. (2014)	15	27.33 to 32.59	-	P300	P300	Still, Sound	36	2	Speller	92	2^†^
	15	27.33 to 32.59	-	P300	P300	Still, Sound	36	2	Speller	87.7	1.65^†^
Breitwieser et al. (2016)	14	20.1 to 32.5	-	SSSEP	tERP	MoTa	2	2	-	44.5	-
Brennan et al. (2020)	30	22.87 to 52.33	-	SSVEP	ET	Strobe	4	1	HA	99.84	24.41
	14	27.7 to 55.5	Brain injury	SSVEP	ET	Strobe	4	1	HA	99.14	15.87
Brunner et al. (2011)	12	23.7 to 28.1	-	MI	SSVEP	Strobe, MoTa	2	2	-	95.6	6.3
Buccino et al. (2016)	15	19.7 to 35.1	-	ME	-	MoTA	4	1	-	82.1	4
Chiarelli et al. (2018)	15	27 to 37	-	MI	-	MoTA	2	1	-	83.28	-
Duan et al. (2015)	3	23 to 27	-	SSVEP	MI	Strobe, MoTa	5	3	Robotic	80	-
	3	23 to 27	-	SSVEP	MI	Strobe, MoTa	5	3	Robotic	73.3	-
Duan et al. (2019)	9	22 to 32	-	SSVEP	MI	Strobe, MoTa	5	1	DVW	87.65	-
Egan et al. (2017)	10	-	-	P300	SSVEP	Strobe	2	2	-	79	-
Fan et al. (2015)	16	21 to 36	-	P300	SSVEP	Still, Strobe	9	2	DVW	99.07	24.19^‡^
	16	21 to 36	-	P300	SSVEP	Still, Strobe	9	2	DVW	98.93	25.95^‡^
Glowinsky et al. (2018)	11	22.3 to 28.9	-	P300	-	Sound	2	1	-	77.43	-
Jalilpour et al. (2020)	6	22 to 27	-	SSVEP	RSVP	Strobe	60	2	Speller	93.06	21.41
Kaongoen and Jo (2017)	10	18 to 22	-	ASSR	P300	Sound	2	2	-	85.33	9.11
Katyal and Singla (2020)	10	19 to 36	-	SSVEP	P300	Strobe	8	2	-	92.3	82.38
Khalaf et al. (2020)	10	23 to 32	-	MI	-	MoTa	2	1	-	83.73	6.82
	11	25 to 32	-	SSVEP	MT	Strobe, Mental	2	2	-	87.46	4.46
Ko et al. (2020)	14	21.49 to 28.11	-	SSVEP	RSVP	Strobe	64	2	Cursor	78.1	7.95
Lee et al. (2018)	20	24 to 32	-	P300	ET	Still	36	2	Speller	98.2	37.6
	20	24 to 32	-	P300	ET	Still	36	2	Speller	97.8	40.6
Li et al. (2018)	6	-	-	SSVEP	MI	Strobe, MoTa	5	1	DVW	91.1	85.8
Lin et al. (2016)	5	22.7 to 24.5	-	SSVEP	EMG	Strobe	60	3	Speller	85.8	90.9
	10	22.9 to 27.5	-	SSVEP	EMG	Strobe	60	3	Speller	82.6	-
Long et al. (2012a)	5	-	-	P300	MI	Spatial	5	3	Cursor	92.84	18.96^‡^
Long et al. (2012b)	11	22 to 32	-	P300	MI	Still, MoTa	5	1	DVW	100	-
Mannan et al. (2020)	20	24 to 46	-	SSVEP	ET	Strobe	48	1	Speller	90.35	184.06
Mousavi et al. (2020)	12	19.4 to 21.4	-	MI	ErrP	Spatial	2	1	Cursor	75.33	2.1
Nann et al. (2020)	8	20.9 to 27.3	-	MI	ET	MoTa	4	3	Robotic	85.89	-
	4	36.6 to 67	Tetraplegic	MI	ET	MoTa	4	3	Robotic	81.25	-
Saravanakumar and Reddy (2019)	10	22.74 to 29.66	-	SSVEP	ET	Strobe	36	1	Speller	98.33	69.21
Shi et al. (2019)	10	19.4 to 22.8	-	MI	ET	MoTa	5	1	DVW	88.43	-
Shin et al. (2018)	18	21.3 to 26.3	-	MI	MT	MoTa, Mental	3	1	-	82.2	4.7
Soekadar et al. (2015)	1	34	Flaccid hand	MI	ET	MoTa	2	2	Robotic	80.65	-
	5	22.7 to 30.3	-	MI	ET	MoTa	2	2	Robotic	76.03	-
Wu et al. (2016)	9	23 to 26	-	SSVEP	P300	Strobe	4	2	-	86.92	24.06
Xu et al. (2014)	11	23 to 36	-	SSVEP	P300	Still	36	2	Speller	93.9	43
Xu et al. (2020)	10	21 to 26	-	SSVEP	P300	Strobe	108	2	Speller	81.67	172.46
	10	21 to 26	-	SSVEP	P300	Strobe	108	2	Speller	79.17	164.69
Yang et al. (2020a)	3	24	-	SSVEP	ET	Strobe	38	2	HA	-	146.67
Yang et al. (2020b)	6	23 to 38	-	MI	SSVEP	Strobe, MoTa	10	2	-	95.53	-
Yao et al. (2014)	11	25	-	MI	SS	MoTa	4	1	-	83.1	-
Yao et al. (2017)	16	22.1 to 26.7	-	MI	SS	MoTa	4	1	-	86.1	-
Yu et al. (2017)	8	23 to 32	-	MI	P300	Still, MoTa	12	1	DVW	-	-
Yu et al. (2019)	18	22 to 32	-	P300	ET	Still	28	2	Speller	93.6	43.8
Zhang et al. (2019a)	6	23 to 26	-	ET	MI	MoTa	11	4	Robotic	93.93	47.41
Zhou et al. (2020)	10	21 to 27	-	SSVEP	ET	Strobe	12	2	Speller	95.42	105.53

† : characters/min;

‡ : s/selection. Underlined elements indicate different experiments within a paper. TSA, tactile selective attention; ET, eye task; tERP, transient event-related potential; MT, mental task; SS, selective sensation; EMG, electromyography; ASSR, auditory steady-state response; MoTa, Motor/Tactile; DVW, Drone/vehicle/wheelchair; HA, House automation; Cursor, Cursor/Game.

**Figure 4:**
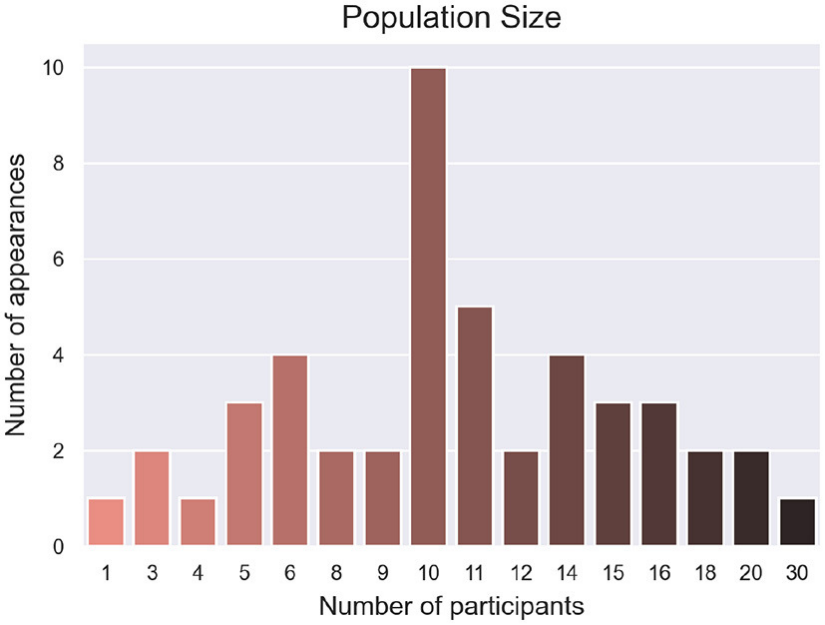
Number of times that the population sizes were used for the validation of hBCI systems.

Only three studies included participants with disabilities. Soekadar et al. (2015) tested the system with one participant with flaccid hand paralysis, a 34 year old male. The study reported that he was able to control a robotic hand *via* motor imagery (MI), even though his accuracy was slightly lower than the average of the other neurotypical participants (76.03% compared to 80.65%). Brennan et al. (2020) included 14 participants with brain injury (although only nine completed the hBCI trials) with an average age range of 41.6±13.9 years. Participants underwent trials with both an SSVEP BCI and an SSVEP Eye Tracker hBCI for comparison. The hBCI trials had higher accuracy than the BCI, with 99.14% compared to 80.26%. Participants with brain injury only did one session of experiments, while neurotypical participants did two. Nann et al. (2020) had four participants with tetraplegia with an average age of 51.8 ± 15.2 years. The study tested an EEG BCI and an hBCI combining EEG with horizontal oculoversion, increasing the accuracy from 58.68 ± 10.62% to 81.25 ± 5.84%. All participants with tetraplegia rated the system as user-friendly and reliable. The study population ranged from 1 to 30 participants, with the most common population size being ten participants (21.3% of the studies), as seen in Figure 4.

**Figure 5:**
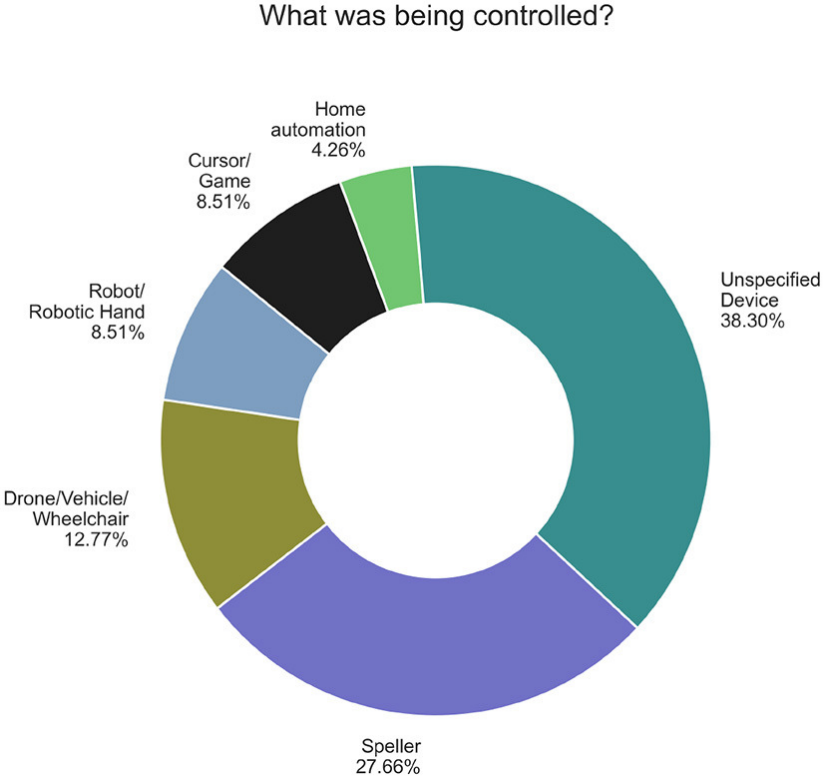
Control tasks distribution.

The devices controlled are represented in Figure 5. Eighteen of the hBCI systems were oriented to control, but only controlled an interface, and did not specify the device. The most common control task was spellers, with 13 systems. Six articles controlled devices that were moved with brain signals such as drones, wheelchairs and other vehicles (physical or simulated). Four controlled robotic devices, four controlled cursors or games, and two focused on home automation systems.

**Figure 6:**
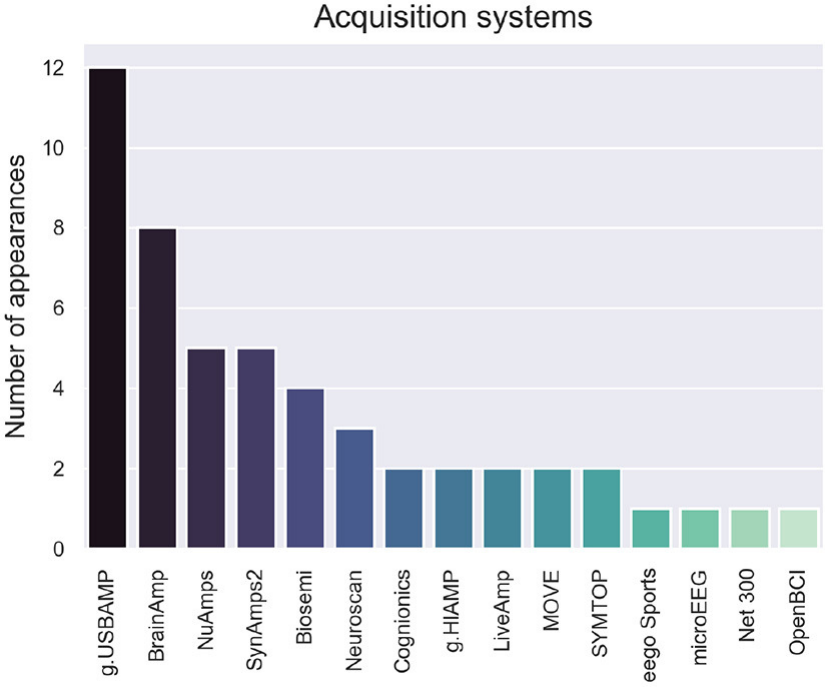
Number of appearances per acquisition system.

Twelve out of 42 of studies used the g.USBamp, as shown in Figure 6. Most of the papers reported using a fabric cap with Ag/AgCl electrodes or golden cups. The only headset-style used was the Cognionics system, used in Yang et al. (2020a,b). Articles that combined multiple acquisition systems were: Buccino et al. (2016) combining the microEEG with the fNIRS NIRScout; Khalaf et al. (2020) combining the g.USBamp with the SONARA TCD; Chiarelli et al. (2018) combining the Net300 with the Imagent fNIRS; Shin et al. (2018) combining Biosemi with LIGHTNIRS; and Glowinsky et al. (2018) combining BrainAmp with ETG-4000 NIRS.

**Figure 7:**
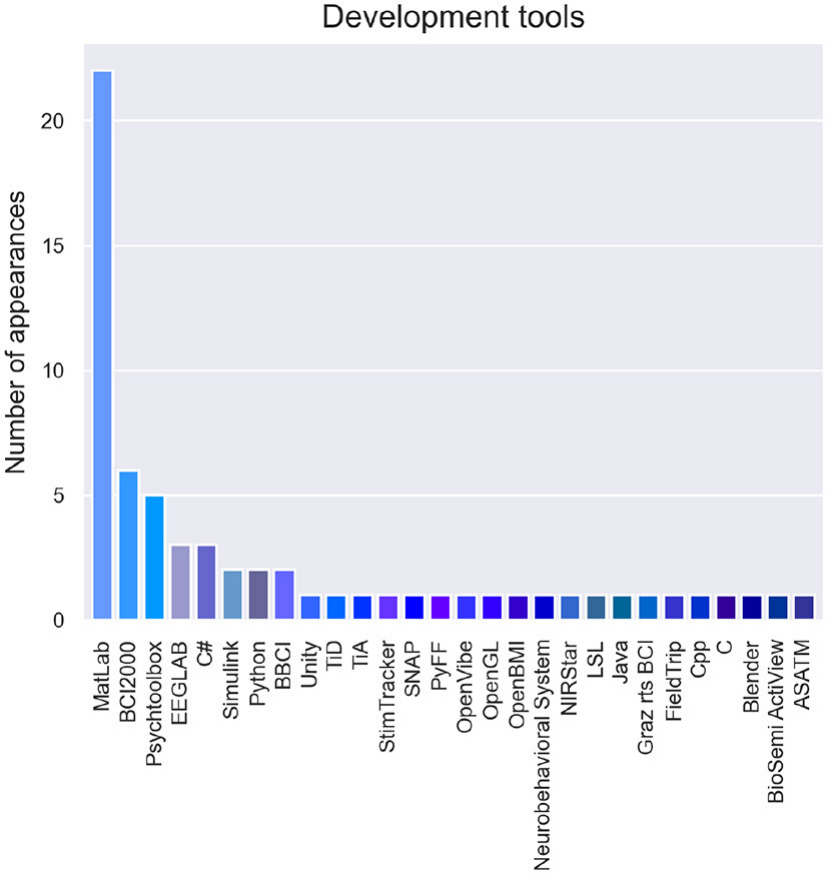
Tools utilized for the development of hBCI systems.

The stated tools that were used for the development of hBCI are represented in Figure 7. Programming languages, toolboxes, stimuli generators and processing tools were considered. The most used language was MatLab, followed by Python and C#, C, and C++. BCI2000, Psychtoolbox, and EEGLAB were the most used toolboxes, usually paired with MatLab.

### 5.2. hBCI taxonomy data

The characteristics according to Choi et al. (2017)'s taxonomy are shown in Table 1. Some papers presented more than one variation of the system. In the 42 papers, 47 systems were presented. Khalaf et al. (2020) presented two systems with different brain signals, one using SMR and one combining SSEP and SCP. Other papers presented synchronous and asynchronous experiments using spellers for cue-based experiments and free-spelling (Lin et al., 2016; Lee et al., 2018; Xu et al., 2020), and others did both sequential and simultaneous stimuli interfaces (Ahn et al., 2014; An et al., 2014). Therefore, all of the following percentages were calculated with 47 total systems, unless otherwise stated.

#### 5.2.1. Diversity of input

**Figure 8:**
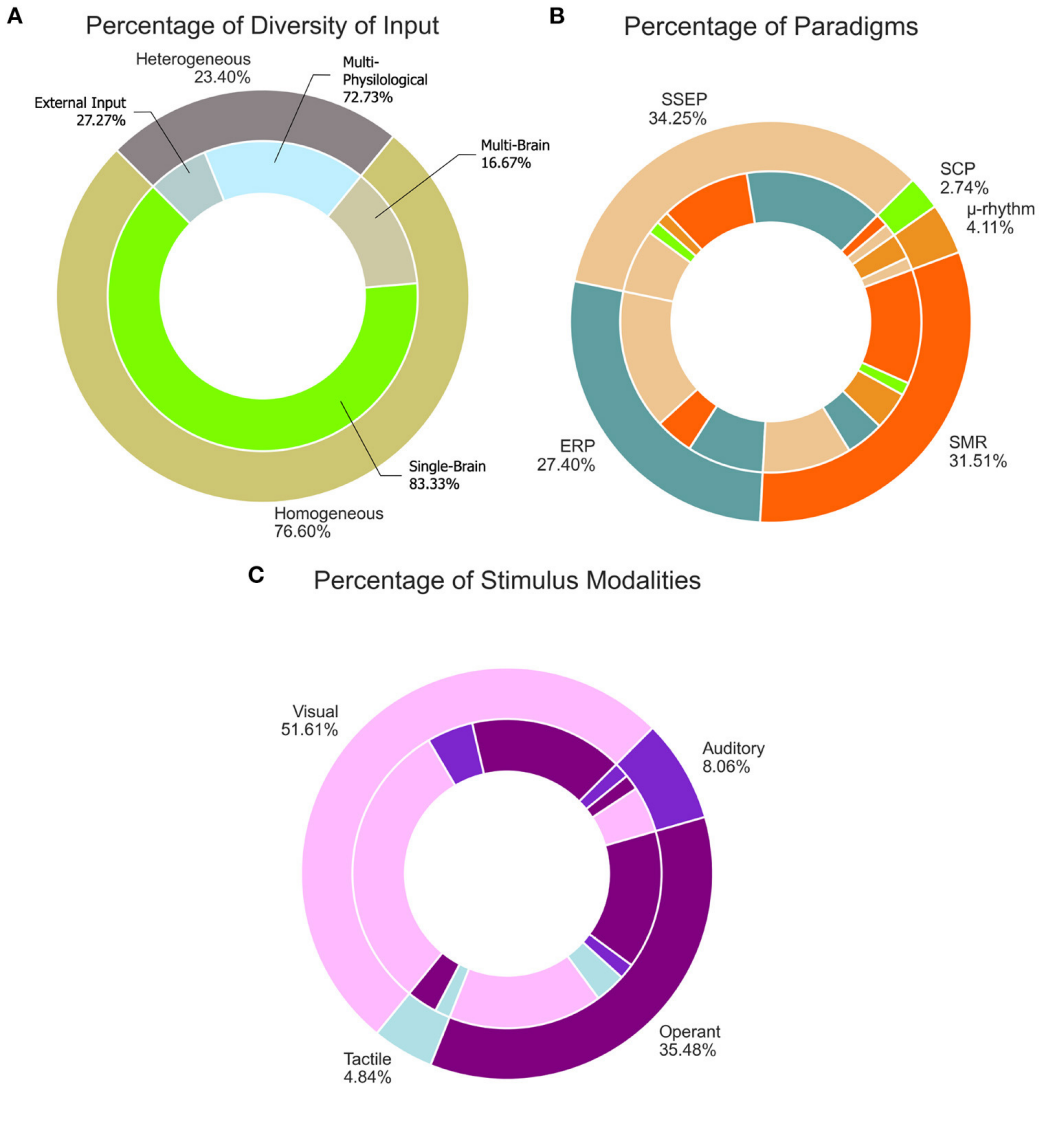
Taxonomy distribution. **(A)** Percentage of diversity of input. **(B)** Percentage of paradigms. **(C)** Percentage of stimulus modalities.

Considering the diversity of input, 36 out of 47 of the systems (76.6%) were homogeneous and 11 (23.4%) were heterogeneous, as show in Figure 8A. Thirty of the homogeneous systems used EEG only and the reminder used a multi-brain signal input approach: two combined EEG and fNIRS, two combined EEG and NIRS, and two combined EEG and fTCD. All the multi-brain signal input systems only presented offline results. Of the eleven heterogeneous systems, eight were multi-physiological and three made use of external input. The multi-physiological signals were mostly EEG and EOG, but Lin et al. (2016) combined EEG and EMG and Zhang et al. (2019a) combined EEG, EOG, and EMG. As for the ones with external input, Mannan et al. (2020) and Brennan et al. (2020) used EEG and an eye tracker and Saravanakumar and Reddy (2019) used EEG and EOG combined with a video-based eye tracker.

#### 5.2.2. Role of operation

Almost 60% of systems were simultaneous in their role of operation, totaling 28 out of 47 systems. Nineteen were sequential, including five sequential-selectors (Long et al., 2012a; Fan et al., 2015; Lee et al., 2018; Ko et al., 2020; Yang et al., 2020a) and two sequential-switching systems (Yu et al., 2017; Li et al., 2018).

#### 5.2.3. Mode of operation

Most experiments relied on cues and used the synchronous mode of operation. Ten had asynchronous modalities, where the participant could self-pace their selections. Zhang et al. (2019a) was the only study to utilize both synchronous and asynchronous. Due to its multi-input nature, Zhang et al. allowed the EOG and EMG to operate asynchronously, and when the EEG mode was selected, the system switched to cue-based operation.

It was also made evident in the review that having mechanisms to amend or confirm selections can increase performance. Mousavi et al. (2020) utilized ErrP to correct MI misclassification, resulting in an improvement in accuracy. Similarly, Soekadar et al. (2015) implemented a task correction with EOG which resulted in a more intentional operation of the system. Fan et al. (2015) implemented a confirmation before the final selection utilizing SSVEP, resulting in one of the highest evaluated accuracies (99.07%).

#### 5.2.4. Mental strategy and brain signature

Almost half of the systems used selective attention (23 systems, 48.9%), about a quarter used operant conditioning (13 systems, 27.7%), and the reminder combined both (11 systems, 23.4%). The selective attention systems were equally divided in terms of paradigms: six used only SSEP, six used only ERP and eleven combined both. As for the operant conditioning, all used SMR. Most were exclusively SMR (10 systems), two combined SMR with μ-rhythms, and one used SMR combined with SCP. The systems with multiple mental strategies mostly used SSEP, and combined it with SMR (6 systems), SMR and μ-rhythm (1 system) and SCP (1 system). The other three combined ERP and SMR.

Figure 8B shows the percentage of brain signatures that were used. The total number for each brain signal signature is represented by the outermost ring, and the combinations made with each signature are represented by the innermost ring. All the systems using μ-rhythms had SMR as well, therefore, the SMR combination is not represented in the figure.

### 5.3. Stimulus modality

Figure 8C shows how the stimulus modalities were distributed for the considered systems. The matching color sections between the inner and outer ring indicate a single stimulus modality. The two most utilized stimulus modalities were visual and operant. Twenty of the 47 systems were purely visual, and 32 systems had visual stimulus combinations. Ten systems were purely operant and 22 of systems combined other modalities with operant stimuli. The combination of both visual and operant was also used, totaling nine systems. Only five systems included auditory stimuli (two purely auditory, two combined with visual and one combined with visual and operant stimuli) and three systems included tactile stimuli (one purely tactile and two combined with operant stimulus).

### 5.4. Interface characteristics

The interface characteristics of the study are shown in Table 2.

#### 5.4.1. Type of targets

Most interfaces used strobic targets. Usually they were associated with SSVEP or RSVP. Jalilpour et al. (2020), for example, combined SSVEP and RSVP to control a speller with 60 targets and achieved an accuracy of 93%. Katyal and Singla (2020) combined SSVEP and P300 to select targets among eight targets separated in two circular sub-groups on the screen. The final offline accuracy was of 92.3%.

Still targets were mostly associated with P300 paradigms. Lee et al. (2018) combined P300 with winking of the eye to control a 6x6 speller, achieving 97.8% in the synchronous mode of operation and 98.2% in the asynchronous. Similarly, Yu et al. (2019) combined P300 and double-blinks to control a 28-character speller. The authors reported 93.6% accuracy.

Spatial targets were associated with MI and required participants to focus to maintain a certain brain pattern. Long et al. (2012b) combined MI and P300, Allison et al. (2012) combined SSVEP and MI to control a 2D-cursor on the screen, and Mousavi et al. (2020) combined MI with ErrP to improve accuracy. Long et al. (2012b) achieved 92.84% while Allison et al. (2012) achieved 60.0% and Mousavi et al. (2020) achieved 75.33% in online trials.

MoTa targets were associated with MI, motor execution, tactile ERP and tactile selective attention, ranging from 44.5 to 93.98% accuracy. Yao et al. (2014), Ahn et al. (2014), and Yao et al. (2017) combined MI with tactile selective attention. Yao et al. (2017) reported 86.1% accuracy offline, Yao et al. (2014) achieved 83.1%, and Ahn et al. attempted a simultaneous and sequential role of operation and achieved 60 and 71%, respectively. Breitwieser et al. (2016) included tactile ERP with SSSEP for an online experiment, but the accuracy was only 44.5%.

Mental targets require applied effort to visualize or do mathematical operations during trials. Only two papers used mental targets, Shin et al. (2018) and Khalaf et al. (2020). Khalaf et al. (2020) combined word formation and mental rotation tasks with SSVEP, achieving 87.46% accuracy. Shin et al. (2018) combined MI and mental arithmetic offline with an 82.2% accuracy. Glowinsky et al. (2018) had 77.43% accuracy for the auditory component. In An et al. (2014), the auditory P300 had a lower accuracy (66.2% at its highest), compared to 85.4% for the visual P300. Kaongoen and Jo (2017) did a preliminary offline study combining auditory steady-state response and auditory P300 with 85.33% accuracy and 9.11 bits/min.

#### 5.4.2. Number of targets

A greater number of targets was mostly seen in spellers. Xu et al. (2020) developed a speller with 108 targets. Twelve 3x3 character matrices were presented to participants at once and they participated in synchronous and asynchronous experiments. Although they had some of the highest ITR (172.5 bits/min for synchronous and 164.7 bits/min for asynchronous) they had the lowest accuracies (81.67% for synchronous and 79.17% for asynchronous) compared to the other spellers, with an average of 90.7%.

#### 5.4.3. Number of steps before selection

Most systems in the review (22 out of 47 systems) had a maximum of two steps. Seven systems utilized three steps and Zhang et al. (2019a) was the only one with four steps. Their system utilized EEG, EOG and EMG modes, each with specific commands. EOG blinking switched modes and a participant might need to make up to four steps to cycle through all the modes and then make a selection.

### 5.5. Effectiveness and efficiency

**Figure 9:**
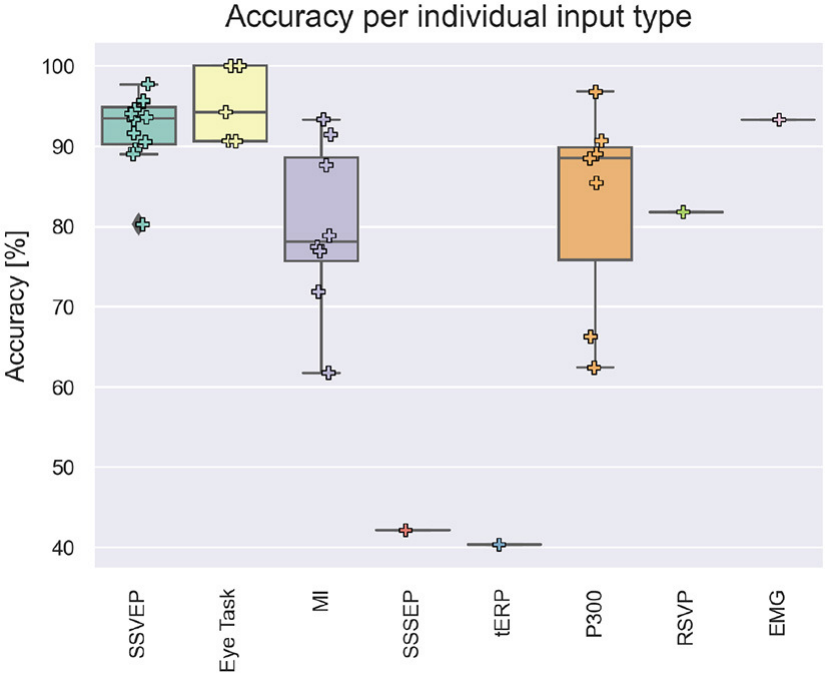
Accuracy per individual input type during online trials.

Table 2 shows the accuracy and ITR results. Sixteen of the 42 studies only performed offline experiments. Some articles also made available the individual input results while in hybrid mode during online trials. Figure 9 shows the accuracy vs. individual input type during online trials. For Figure 9, when multiple conditions were tested (e.g., results with different number of runs, with and without correction mechanisms, with more or less samples, etc.), the best results were considered. In papers where both real-world and simulation results were presented, only the real-world control application results were considered. Eye gaze and EOG activities (blinking, frowning, vertical/horizontal movements, etc) were reported as eye-tasks.

**Figure 10:**
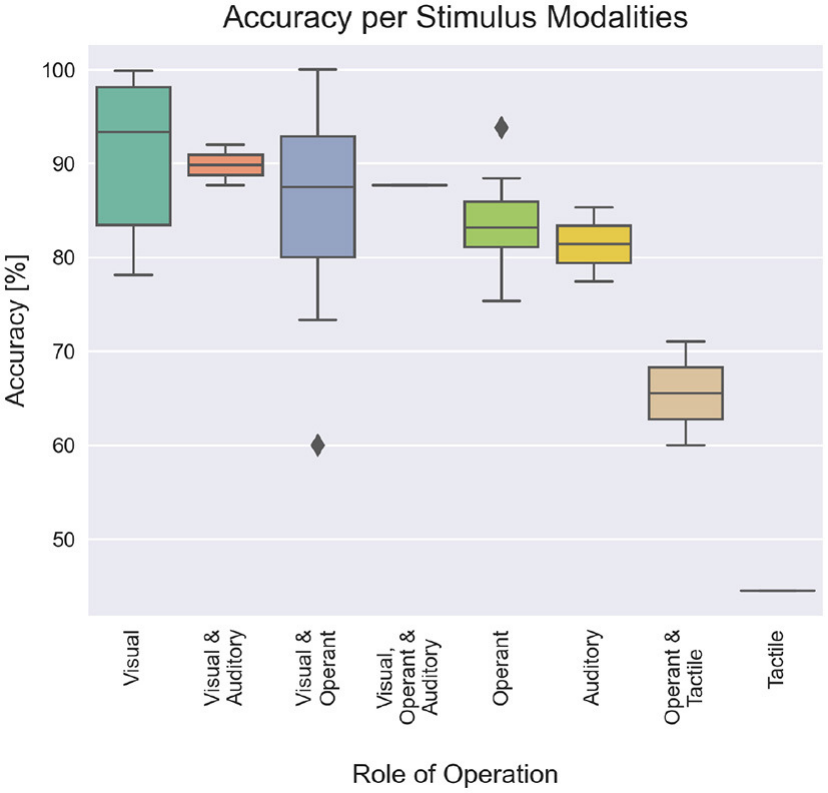
Average accuracy per stimulus modality.

Figure 10 shows the relationship between accuracy and each stimulus modality used in the studies. Although not all modalities had the same sample size, we can see a trend where systems using visual pathways have higher accuracies than the others.

## 6. Discussion

As described by different research groups, the implementation of hBCI can be a bigger challenge for children than for adults. This review sought to extract and analyse existing hBCI applications, and explore what might be the most appropriate approaches for children. Below we present some considerations based on the extracted factors that might be helpful in the design and experimentation of future interfaces that are easier to use and more adapted for children.

### 6.1. Headsets and caps

Some authors have written that children report discomfort when using a BCI cap or headset (Zhang et al., 2019b; Jadavji et al., 2022). Headsets are, presumably more comfortable and easier to don than caps, but only one of the articles in this review utilized a headset in the hBCI system Yang et al. (2020a,b). Hybrid-hBCI systems that combine multiple input caps (Chiarelli et al., 2018; Shin et al., 2018; Khalaf et al., 2020) could be even more uncomfortable due to the overlapping caps/headsets. New approaches to headset/cap designs so they can be more comfortable are needed. Additionally, headsets with a built-in capability of measuring different brain signal inputs may be preferable for use with children.

### 6.2. Diversity of input

Having an external input can facilitate selection if the participant does not have significant impairments. Inputs such as eye trackers, joysticks and switches add reliability to the system and therefore increase its performance. Three of the selected papers used external inputs, Mannan et al. (2020) and Brennan et al. (2020) used NIR eye trackers and Saravanakumar and Reddy (2019) utilized a camera-based eye tracker. All the systems used the eye gaze as a means to narrow down the possible targets. Mannan et al. (2020) and Saravanakumar and Reddy (2019) used the gaze to select the character sub-group and the SSVEP to select the character within the sub-group while Brennan et al. (2020) used the gaze to select the sub-region on the screen and compare it against the SSVEP selection for the final decision. Their accuracy results were among the highest (Brennan et al., 2020 with 99.84%, Saravanakumar and Reddy, 2019 with 98.33% and Mannan et al., 2020 with 90.35%), even when the system was utilized by nine participants with brain injury (Brennan et al., 2020 with 99.14%).

### 6.3. Role of operation

Multi-tasking generally decreases processing speed and increases the amount of information needed to make a decision (Howard et al., 2020). Detecting multiple brain signals simultaneously can make it easier for a participant since they only have to focus on one thing and it can decrease mental fatigue, especially for children (Cowan et al., 2006). Ahn et al. (2014)'s two experiments combining MI and tactile selective attention, with sequential and simultaneous roles of operation, yielded 71% accuracy for sequential, while the simultaneous reached 60%. Thus, multi-tasking reduced accuracy in this task.

The systems that implemented confirmation/correction mechanisms had beneficial results (Fan et al., 2015; Soekadar et al., 2015; Mousavi et al., 2020). We infer that children could take advantage of this feature when using hBCI. The ErrP can be of great assistance especially for the children that are getting started with hBCI systems as it is a natural response that does not require any training nor extra action.

### 6.4. Mental strategy and brain signature

Switching between brain signatures or performing multiple brain signatures simultaneously can increase the complexity of the system, especially if the brain signatures belong to different mental strategies. For example, Duan et al. (2015) utilized SSVEP to move a robot, mu-rhythms to switch modes, and MI for grasping. It was the only system with more than two brain signals for control. Its accuracy, 73.3%, was lower than the average of all the included papers, which was 85.64%. Similarly, but in a simultaneous role of operation, Allison et al. (2012) developed a system where a ball could be moved in a 2D space utilizing SSVEP and MI for horizontal and vertical movement, respectively. The average accuracy was 60%.

### 6.5. Stimulus modality

Most papers chose visual pathways to stimulate the brain. Visual stimulation is the most used, and it is also the least complex modality. Visual paradigms, in general, elicit clear signals over the occipital and parietal regions, especially when using SSVEP and P300 (de Haan, 2007; Ehlers et al., 2012). On the other hand, operant modalities require a certain level of training and focus from the participant to generate distinguishable signals (Yuan and He, 2014). The auditory modality was considered more complex than the operant modality because it requires more attention and has a steeper learning curve than operant modalities (Nijboer et al., 2008). Lastly, tactile modalities require body awareness, and can become confusing with multiple targets (Brouwer and van Erp, 2010).

We suggest that modalities for children, from the easiest to the hardest, based on the accuracies per modality seen in Figures 9, 10, would be visual, operant, auditory and tactile. There were not enough studies that used auditory and tactile modalities to statistically confirm their lower performance compared to visual and operant. However, the studies included in our scoping review presented accuracies and ITR below the average of visual and operant modalities. We can also infer that both auditory and tactile modalities would require a higher level of auditory perception and body awareness, which might not be well-developed in some children.

Having a system that requires the engagement of multiple senses through multiple stimulus modalities might also increase the system's complexity when stimulus modalities work in parallel to each other (i.e., selecting different targets). An et al. (2014) experimented with both roles of operation combining visual and auditory P300. During the sequential operation experiment, stimuli alternated between visual and auditory stimuli within 300 ms, so that two independent decisions could be made in parallel (selecting the sub-group and the character within sub-group). When asked about the workload, participants reported that the sequential modality had considerably higher workload than the experiments in which they used each paradigm individually. Allison et al. (2012) combined visual (SSVEP) and operant (MI) stimulus modalities to move a cursor in a 2D space. Although no workload assessment was done, the average accuracy across participants was 60%.

On the other hand, when multiple stimulus modalities are combined to reinforce the selection of the same target, the complexity can be diminished. During the simultaneous operation experiment, An et al. (2014) organized visual and auditory stimuli so that both stimuli referred to the same target. The reported workload for the simultaneous operation experiment was lower than the individual paradigm experiments. Most participants felt more relaxed during the simultaneous experiment as they could subconsciously switch between modalities to avoid increased mental demand as both stimuli were redundant. Khalaf et al. (2020) combined visual (SSVEP) and operant (SCP) modalities to make a selection. During the experiment, participants had two targets with different SSVEP flashing frequencies, each associated with either a mental rotation or a word formation task. This allowed participants to only focus on one target whilst still reinforcing their choice. The average accuracy across participants was 87.46% (result averaged from each individual task).

### 6.6. Target types

Seo et al. (2019) showed that certain types of targets can cause more fatigue in users. The authors show that, for example, SSVEP has a higher eye-fatigue level than P300. Based on qualitative comments reported in the article and the reported accuracies, we infer that some target types require less workload than others, and therefore, we might want to consider the easier ones to use with children. The complexity order of targets that we propose, from less to more complex, considering the expected required effort and fatigue, would be strobic targets, still targets, spatial targets, motor/tactile targets, mental tasks, and sound cues.

Most of the qualitative comments in the articles were regarding systems utilizing SSVEP stimuli. Allison et al. (2010), Brunner et al. (2011), Allison et al. (2012), and Mannan et al. (2020), reported low annoyance for strobic targets. Figure 9 shows that the SSVEP paradigm had the highest accuracy average, followed by the P300. It is possible that children might be more annoyed by the flashing than adults, but current research shows that children perform well using SSVEP Norton et al. (2018). It is also important to consider that some frequencies between 12 and 25 Hz may induce seizure in children with photosensitivity (Fisher et al., 2005; Okudan and Ozkara, 2018).

We assume spatial targets could be the most engaging for children as it is easy to add graphical elements with attractive and game-like designs, but it can also be complex because spatial targets require more training and can result in lower accuracies as seen in Allison et al. (2012) and Mousavi et al. (2020).

MoTa target types, as well as all the other off-screen targets, can be especially beneficial for vision-impaired children. Nonetheless, the studies involving tactile stimuli had accuracies below 90% and only one study reported online results (Ahn et al., 2014; Yao et al., 2014, 2017; Breitwieser et al., 2016).

One weakness of mental task experiments is that they have a low ITR [4.7 bits/min for Shin et al. (2018) and 4.46 for Khalaf et al. (2020)]. Plus, it could be difficult for children to maintain their interest since it involves focusing, potentially on tasks that might not seem playful. None of the studies had online trials, preventing the assessment of expected performance in real-world scenarios.

Finally, auditory cues produced a lower accuracy than visual targets (An et al., 2014; Glowinsky et al., 2018). Although it could also be an alternative for children with impaired vision, An et al. (2014) reported a higher workload for off-screen stimuli when comparing visual and auditory P300. This is consistent with how auditory cues have been reported to be more difficult to learn (Nijboer et al., 2008).

A sequential role of operation would be recommended for a pediatric hBCI as it only requires focus on one stimuli at a time rather than multi-tasking. To compensate for their shorter attention span, as reported by Riccio et al. (2013), the system could use a singular visual stimulus modality. Lastly, to attenuate the system's complexity, a single brain signal signature can be used with the help of an external input to help increase the system's accuracy.

## 7. Limitations

The authors of this paper only considered EEG-based systems as they are more commonly used, but hybrids using other brain signals exist. Many articles were excluded from this review for various reasons: the word choices for the search terms may have missed some studies or techniques if authors used different terms to define hybrid-related concepts; the necessary information to be included in this review was missing; the hybrid aspect did not meet our inclusion criteria (e.g., the eye input was used only as a switch or the brain component was a secondary input). We also acknowledge that the sample size for statistical assumptions is small and some of our conclusions could be skewed due to the uneven number of studies per feature and uneven number of trials for each studies. We also did not specifically use criteria based on empirical evidence for what might be important to consider for children using hBCI as we could not find such studies with the pediatric population. Future research might prove that some of the inferences made in this paper were inaccurate.

## 8. Conclusion

This scoping review analyzed 42 papers that presented 47 different hBCI systems. Articles were focused on clinically viable hBCI that were EEG-based and had hybrid inputs or brain signals for the purpose of improving system performance. Using a taxonomy for categorization of features and other interface traits, we inferred how systems may be more or less complex, for users in general, and for children. Such considerations were based on accuracy and ITR results, and also qualitative comments presented in the studies.

We conclude that hBCI systems that have a single brain signal signature and external input, using a sequential role of operation with a singular visual stimulus modality, should have a lower complexity than other combinations. Additionally, interfaces using from two to five (or less than 37 for spellers) strobic targets, with single or double steps before selection, can also attain good performance while keeping the system simple. The inferences made throughout this paper could serve as a guideline for future researchers that are developing hBCI for children.

## Author contributions

MM and KA contributed to the concept, design of the study, and filtered the papers. MM ran the database searches, organized the studies, created the graphs, and wrote the drafts for the manuscript. KA revised the manuscript. Both authors contributed to manuscript revision, and read and approved the submitted version.

## Funding

This research was funded by the 2019 Alberta Innovates SPOR Graduate Studentship competition, the SMART Network Innovation Fund and the Glenrose Rehabilitation Hospital Foundation.

## Conflict of interest

The authors declare that the research was conducted in the absence of any commercial or financial relationships that could be construed as a potential conflict of interest.

## Publisher's note

All claims expressed in this article are solely those of the authors and do not necessarily represent those of their affiliated organizations, or those of the publisher, the editors and the reviewers. Any product that may be evaluated in this article, or claim that may be made by its manufacturer, is not guaranteed or endorsed by the publisher.
