# Supplementary figures and images for: EEG hybrid brain-computer interfaces: A scoping review applying an existing hybrid-BCI taxonomy and considerations for pediatric applications
Source: Front Hum Neurosci. 2022 Nov 17;16:1007136. doi: 10.3389/fnhum.2022.1007136 (PMC9715435; doi:10.3389/fnhum.2022.1007136)

# Accuracy per Number of Commands

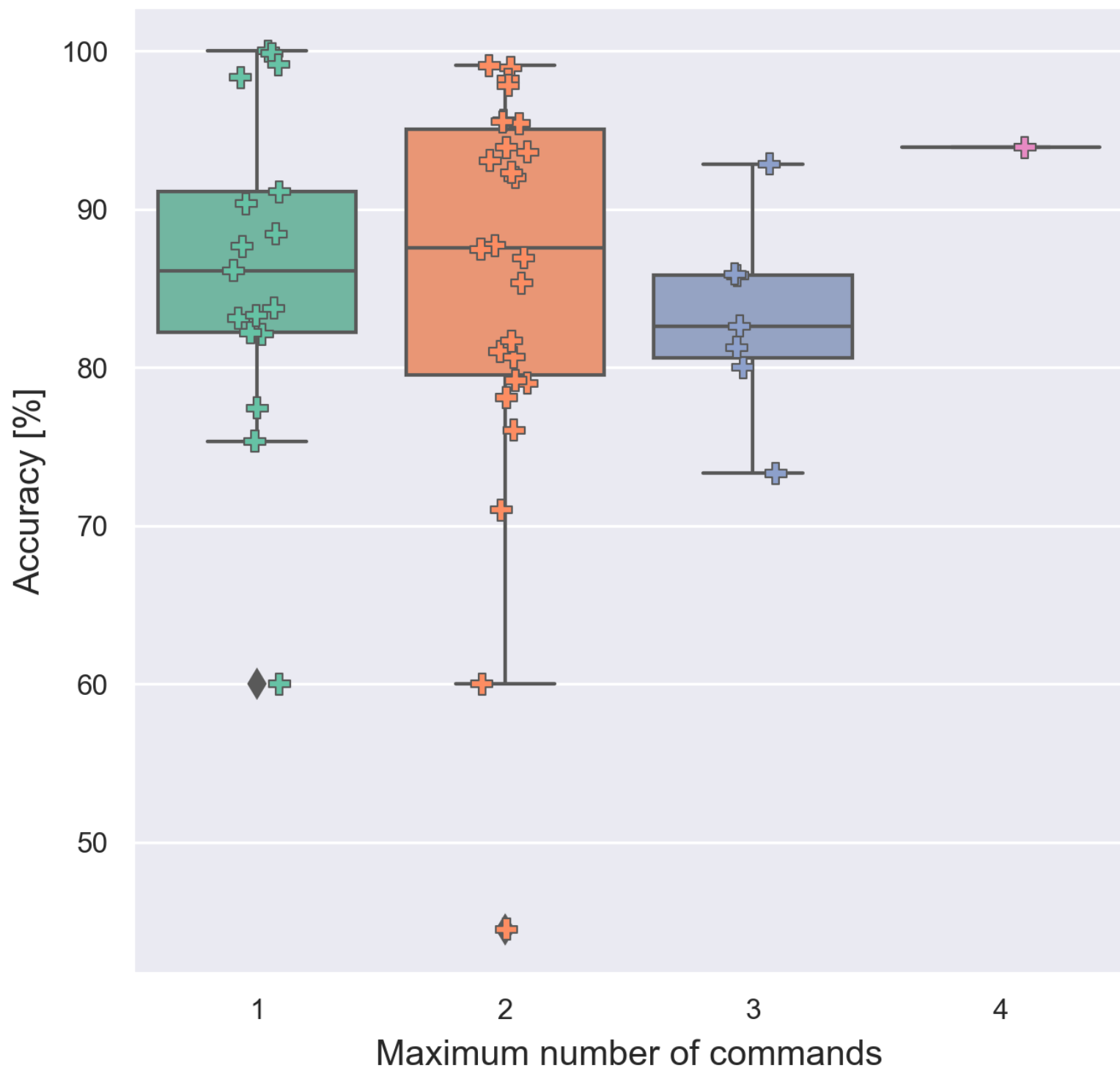

Supplement: Supplementary file 1 [file Data_Sheet_1.PDF]
